# Lung Ultrasound in Coronary Care Unit, an Important Diagnostic Tool for Concomitant Pneumonia

**DOI:** 10.3390/diagnostics12123082

**Published:** 2022-12-07

**Authors:** Costantino Mancusi, Ilaria Fucile, Paola Gargiulo, Mariangela Mosca, Biagio Migliaccio, Christian Basile, Giuseppe Gargiulo, Ciro Santoro, Carmine Morisco, Nicola De Luca, Giovanni Esposito

**Affiliations:** 1 Emergency Medicine School, Department of Advanced Biomedical Sciences, University of Naples Federico II, 80131 Naples, Italy; 2 Division of Cardiology, Department of Advanced Biomedical Sciences, University of Naples Federico II, 80131 Naples, Italy

**Keywords:** pneumonia, coronary care unit, chest X ray, chest CT scan

## Abstract

Background: In the setting of a coronary care unit (CCU), the early detection of pneumonia is of paramount important to prevent severe complications. This study was designed aiming to evaluate the diagnostic accuracy of lung ultrasound (LUS) in the detection of pneumonia and compared with chest X-ray (CXR). Method: We enrolled 110 consecutive patients admitted to the CCU of Federico II University Hospital. Each patient underwent CXR and bedside LUS on admission. The final diagnosis (pneumonia vs. no pneumonia) was established by another clinician reviewing clinical and laboratory data independent of LUS results and possibly prescribing chest contrast-enhanced CT (*n* = 34). Results: The mean age was 70 ± 11 years old, and 68% were males. Pneumonia was clinically diagnosed in 26 (23%) patients. LUS was positive for pneumonia in 24 patients (sensitivity 92%, specificity 81%). Chest radiography was positive for pneumonia in nine patients (sensitivity 43%, specificity 95%). Using CT scan as a reference, LUS exhibited 92% sensitivity and a specificity of 96%. In ROC curve analysis, the diagnostic accuracy of CXR and LUS for the diagnosis of pneumonia was 0.86 (95% CI 0.77–0.94), which was higher than CXR 0.68 (95% CI 0.55–0.84), *p* < 0.05. Conclusion: Based on the findings of the present study, the accuracy of LUS in the detection of pneumonia was significantly higher than chest X-ray with comparable sensibility to CT scan.

## 1. Introduction

In the setting of coronary care unit (CCU), the early detection of pneumonia is of paramount importance to prevent severe complications in patients affected by acute coronary syndrome or acute heart failure. Although the diagnosis of pneumonia is based on clinical data, the performance of imaging modalities is necessary to confirm the diagnosis [1]. Chest X-ray (CXR) is the most commonly performed test. However, it has significant limitations such as patient exposure to ionizing radiation, relatively low sensitivity in detecting pulmonary inflammatory lesions and interpretation discrepancies among specialists [2]. The chest CT scan is considered the “gold standard” for the detection of pneumonia and other pulmonary lesions, but it cannot be used as a first-line radiological examination in all patients with suspected pneumonia [3]. This is mainly because it is often costly, not always available and it involves a high radiation dose. Moreover, patients need to be transferred to the radiology department. Lung ultrasound (LUS) can be advantageous because is a radiation-free imaging modality, which makes it useful also for daily monitoring and might be performed at the bedside, reducing the need to move the patient [4,5]. LUS has been tested in the setting of emergency department (ED), demonstrating it to be superior to CXR for the diagnosis of pneumonia [4]. In the setting the CCU, its use was mainly related to the quantification of lung congestion in patients with acute or chronic heart failure [6].

The aim of the present study was to evaluate the diagnostic accuracy of bedside LUS in the diagnosis of acute pneumonia in comparison with a CXR in the setting of CCU.

## 2. Materials and Methods

From January 2021 to June 2022, we enrolled 117 patients admitted to the CCU of Federico II University Hospital. All patients underwent LUS and CXR. A subgroup of 34 patients also performed a chest CT scan as clinically indicated.

The discharge diagnosis of pneumonia was made by 2 physicians outside the study group, who could access the data obtained in the whole clinical folder, including radiological (CXR, LUS and chest-CT scan), microbiological diagnostics investigations (sputum and blood cultures, and antigen tests) and whole biochemical tests performed during the hospital stay. The diagnosis of acute pneumonia was made combining patient’s clinical presentation (including new onset of systemic features at least one among: sweat, chills, aches and pain, temperature ≥38 °C or <36 °C and symptoms of an acute lower respiratory tract illness at least one among: cough, sputum production, dyspnea, chest pain, altered breathing sounds at auscultation, and chest imaging according to current guidelines) [7,8]. In total, 117 patients were analyzed by the 2 independent and blinded physicians with a concordance of 0.94 (k-Cohen 0.841), of which 7 patients were then excluded due to disagreement on the final diagnosis. Thus, the final study population consisted of 110 patients. 

Concomitant heart failure was diagnosed according to current guidelines.

LUS images have been acquired with a 5–9 MHz convex probe. We followed a standardized protocol for LUS performed in 6 scans for each hemi thorax (anterior, lateral and posterior scanning zones, divided by anterior and posterior axillary line, and upper and lower regions), covering 12 imagine regions, in all the patients in the supine position, at rest. If feasible, a posterior scan was achieved with the patient in the seated position for a better visualization of posterior recesses [9]. LUS was performed in the supine position only when forced decubitus was present (i.e., severe vertebral disease, severe trunk muscular stiffness or non-collaborating patient). In this case, the bed headboard was lifted between 30 and 45 degrees for anterior–lateral scans, and the patient was turned into lateral decubitus for posterior scans. The examinations were performed by experienced residents in emergency medicine (IF, MM, BM) and reviewed offline by one cardiologist physician (CM) with a long experience in LUS. Residents involved in the study have Level 2 EFSUMB competence (>300 LUS performed with more than 2 years of experience), while CM has Level 3 EFSUMB competence (full competence in LUS with teaching, research and development as ‘expert’ in the field). 

**Figure 1:**
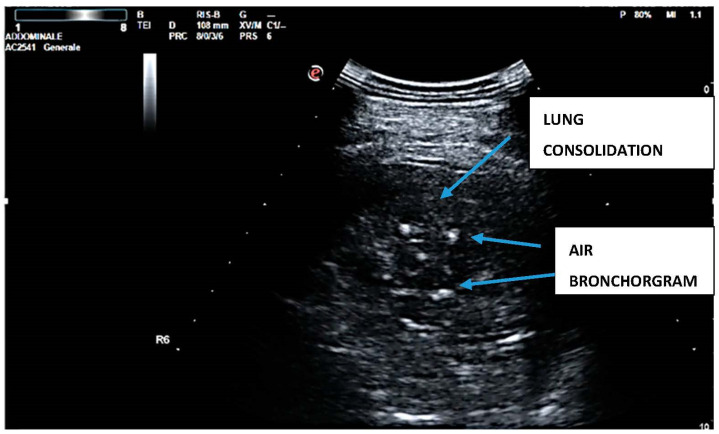
Lung consolidation.

LUS examination was considered to be positive if signs of sonographic consolidation and/or focal multiple B-lines were observed. ‘Sonographic consolidation’ is defined as a small sub-pleural hypoechoic region or large hypoechoic region with liver- or tissue-like echotexture (Figure 1) [10].

CXR was performed in only one setting (anterior–posterior in semi-supine decubitus) and read blind by a radiologist. Patients with uncertain diagnosis underwent chest CT scan with or without intravenous contrast media based on clinical judgment.

To assess the reproducibility of LUS for the identification of lung consolidation, a sample size of 20 examinations was analyzed blind by two expert operators in LUS (CM and CS) with a concordance of 0.95 (k-Cohen 0.86).

The primary end point was to evaluate the sensitivity and specificity of LUS and CXR performances to correctly detect pneumonia. All patients gave their informed consent to be included in the study. The study was conducted in accordance with the principles of the Declaration of Helsinki.

The sample size was calculated based on the literature to find a difference of 30% in sensitivity between LUS and CXR; with a power of 90% and an alpha risk of 0.05, the required number of patients would be 100.

Data were analyzed using SPSS version 24.0 (SPSS, Chicago, IL, USA). Continuous data are expressed as mean ± standard deviation and categorical variables are expressed as percentages. Quantitative variables were compared by using Student’s *t*-test, while chi-square distribution was used to compare categorical variables. A *p*-value < 0.05 was considered statistically significant. The population was divided into two groups: patients with acute pneumonia and patients without acute pneumonia. The diagnostic performance of LUS and of CXR was assessed by calculating sensitivity, specificity, positive and negative predictive value, positive and negative likelihood ratio. The same analysis was repeated and divided into patients based on the presence of absence of pleural effusion. In a subgroup of patients who underwent chest CT scan, the diagnostic accuracy of LUS and CXR was compared to the chest CT scan. Areas under the curve (AUC) and receiver operating characteristic (ROC) curves were used to compare the performance of the different diagnostic tests (CXR, LUS and chest CT scan). Further analyses were performed to analyze the impact of combined LUS and C-reactive protein for the diagnosis of acute pneumonia considering the test positive if patients had positive C-reactive protein and LUS. The Hs-C-reactive protein was defined as positive if >5 mg/dL based on previous findings [8].

To assess the accuracy of LUS for the diagnosis of lung consolidations when compared to chest CT scan sensitivity, the specificity and accuracy of LUS were calculated considering the chest CT scan as a gold standard.

## 3. Results

The mean age was 70 ± 11 years old, and 68% were male. The main reasons for admission in CCU were acute coronary syndrome, acute heart failure and severe valvular heart disease. Pneumonia was clinically diagnosed in 26 (23%) patients, and 54.8% of the study participants had pleural effusion, which was bilateral in 48.1%. Table 1 summarizes the baseline characteristics of the patients who have or did not have acute pneumonia. Patients with pneumonia had a higher hs-C reactive protein level (*p* < 0.001) and a lower PaO_2_/FiO_2_ value (*p* < 0.048) than patients who did not have pneumonia. Pleural effusion was more common among patients with pneumonia (*p* < 0.0001).

LUS correctly detected pneumonia in 24/26 patients, demonstrating a sensitivity of 92%. CXR was positive in 9/26 patients with pneumonia, yielding a sensitivity of 43%. The specificity of LUS and CXR were, respectively, 80% and 95% (Table 2).

The same analysis was repeated, and we divided the study population based on the presence (*n* = 55) or absence (*n* = 55) of pleural effusion on LUS examination. Results are reposted in Table 3.

The presence of pleural effusion significantly reduced the diagnostic accuracy of both LUS and CXR.

The sensitivity and specificity for the chest CT scan was calculated in a subgroup of 34 patients who performed the examination based on clinical indication with highest sensitivity and specificity. Table 4 compares patients with all available diagnostic tests (i.e., LUS, CXR and CT scan).

Further analysis was performed combining positive LUS with high C reactive protein for the diagnosis of acute pneumonia. The sensitivity, specificity and accuracy were, respectively, 92% (95% CI 74–99), 88% (95% CI 78–94), and 89% (95% CI 81–94).

**Figure 2:**
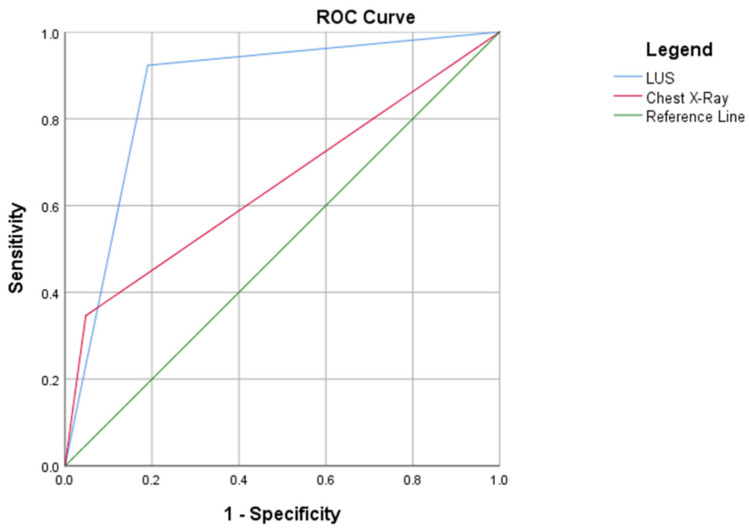
AUC of LUS and CXR for the diagnosis of acute pneumonia.

A first ROC curve analysis was built comparing the diagnostic accuracy of CXR and LUS for the diagnosis of pneumonia. The AUC for LUS was 0.86 (95% CI 0.77–0.94), which was higher than CXR (AUC 0.68, 95% CI 0.55–0.84), *p* < 0.05, as shown in Figure 2.

**Figure 3:**
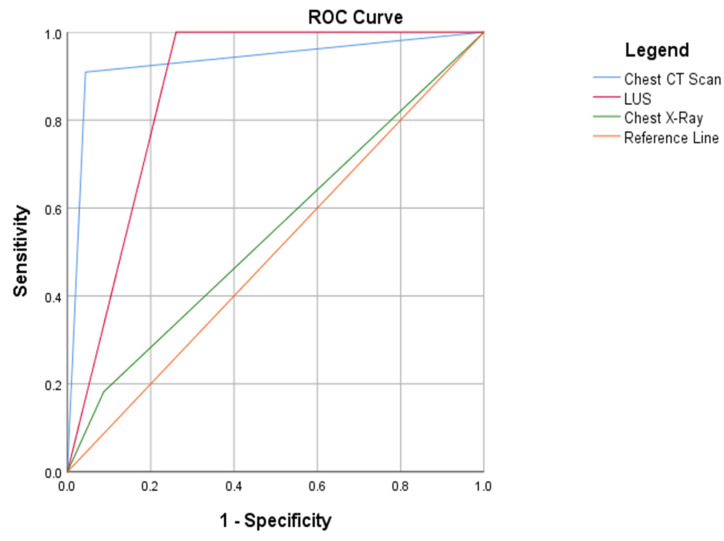
AUC for LUS, CXR and chest CT scan for the diagnosis of acute pneumonia.

A second ROC curve analysis was then built in the subgroup of 34 patients who also underwent a chest CT scan. In this subgroup of patients, pneumonia was diagnosed in 12 patients (33%). The chest CT scan reported in Figure 3 has the highest accuracy for the diagnosis of pneumonia (AUC 0.93, 95% CI 0.79–1.00), which was not statistically significantly higher than LUS (AUC 0.86, 95% CI 0.74–0.99). The accuracy of CXR was suboptimal (AUC 0.57, 95% CI 0.33–0.80).

Finally, the diagnostic accuracy of LUS for the diagnosis of lung consolidation was assessed compared to chest CT scan, as reported in Table 5.

## 4. Discussion

Our study demonstrated that LUS is an important diagnostic tool helping in the diagnosis of pneumonia in patients admitted in the CCU, showing excellent sensitivity and specificity with a slightly lower accuracy than chest CT scan. This finding expands previous reports on the role of LUS in critical care [11,12,13], emphasizing its importance in the evaluation of pleural effusion and extravascular lung water in acute pulmonary edema [14,15] and in monitoring the acute decompensated heart failure compared to N-terminal pro-brain-type natriuretic peptide treatment [16,17].

In a Chinese study involving patients admitted to the ED, Liu et al. reported that the sensitivity of LUS significantly outperforms chest X-ray (94.6 versus 77.7%, *p* < 0.001) with a significantly higher accuracy (96.1% versus 83.8% (*p* < 0.001)) using chest CT scan as the gold standard [18]. Chavez et al. [19] reported in a systematic review and meta-analysis that included 10 studies the diagnostic accuracy of LUS for the diagnosis of pneumonia in ED and ICU in patients >18 years of age. These studies provided a combined sample size of 1172 participants. They found that LUS had a high sensitivity (94%) and specificity (96%). In the setting of a regular geriatric ward, the LUS demonstrated higher accuracy than CXR when compared as the gold standard with the final clinical diagnosis of pneumonia, with comparable values of accuracy (90%) as in our study [20].

These results indicate that point-of-care LUS has the potential to be an initial imaging modality for the diagnosis of pneumonia, as it emerged that the accuracy of LUS was significantly higher than CXR and comparable to a chest CT scan. Therefore, considering characteristics such as safety, low cost, being portable, and being available, ultrasonography seems to be a reasonable tool for the screening and diagnosis of patients with pneumonia requiring acute cardiac care. Compared with chest CT scan, LUS has a sensitivity of 100%, allowing to definitively rule out acute pneumonia if the examination is negative. This result is not surprising given the results of a bigger study performed in the setting of emergency department by Nazerian et al. [21]. However, it should be underlined that in the presence of central foci of pneumonia, not reaching the pleura, LUS is not able to visualize the lung consolidation, and thus, sensitivity of 100% might not be a fully reproducibly result.

In the setting of the CCU, LUS has been tested as a diagnostic tool for the assessment of lung congestion mainly in patients with acute heart failure and acute coronary syndrome [22,23]. Acute pneumonia is a frequent comorbidity in patients admitted with acute coronary syndrome and often an unrecognized cause of dyspnea [24]. This underlined the need for an affective chest diagnostic modality, considering the difficulties in moving patients to the radiology ward due to hemodynamic instability and potential life-treating arrhythmia. In this regard, our study is the first to assess the diagnostic performance of LUS in diagnosing acute pneumonia in the setting of CCU, confirming the good diagnostic performance of LUS as reported in the general intensive care unit [25,26]. The importance of performing LUS to confirm the diagnosis of pneumonia has been recently tested in patients admitted in the emergency department by Javoudin et al., showing that LUS was a powerful tool to improve Community-Acquired Pneumonia diagnosis in the ED, reducing diagnostic uncertainty from 73% to 14% [27]. This might be particularly useful in the setting of CCU where diagnostic uncertainty in patients with concomitant heart failure, acute coronary syndrome and pneumonia might challenge the in-charge cardiologist. Of course, we need to underline the important limitations of LUS regarding its inability to visualize deep pulmonary lesions that are not in contact with the pleura [28]. Moreover, in some cases, LUS may also be limited by the impossibility to examine accurately the whole chest, particularly in patients in critical conditions such as those with recent acute heart failure of acute ST elevation myocardial infarction. The presence of pleural effusion significantly reduces the diagnostic accuracy of LUS and CXR, which is probably because of the confounding imaging due to the presence of compressive lung atelectasis. However, due to the low number of patients in the sub-analysis, further studies are needed to demonstrate the impact of pleural effusion on the diagnostic accuracy of LUS.

The addition of C-reactive protein to LUS improves diagnostic accuracy, leading to a substantial improvement in the specificity of the diagnostic test (81% vs. 88%). This is of particular importance due to the avoidance of false positive results and thus unnecessary antibiotics treatments. Previous findings have demonstrated that the combination of LUS with pro-calcitonin (PCT) level improves diagnostic accuracy in the setting of ED and for the diagnosis of ventilator-associated pneumonia in the setting of intensive care unit [29,30]. In both studies, the accuracy of LUS combined with PCT was comparable to that obtained in our study combining LUS and C-reactive protein. A recent study performed in an acute Geriatrics Unit demonstrated that adding clinical and biochemical variables (including C-reactive protein) to LUS improved diagnostic accuracy, leading to a more accurate diagnosis of pneumonia in old patients with acute respiratory failure [31]. Thus, our findings suggests that LUS is a useful tool for the diagnosis of acute pneumonia in CCU and is accuracy improved combining information from serum C reactive protein mainly reducing the number of false positive results and increasing the specificity of the test. Finally, it emerged that patients with pneumonia have a lower PaO_2_/FiO_2_ compared to patients without pneumonia showing a greater lung involvement with a reduced area of ventilation in the lungs and thus compromised blood gas exchange.

Our study has some limitations. First, the population sample is limited. A larger study population is needed to confirm our results. Nevertheless, the study’s results are significant even with this sample size, although larger samples would have probably reduced confidence intervals. Unfortunately, PCT levels were not available in all our patients and thus could not have been tested as a combined variables with LUS. Only one-third of the patients performed a chest CT scan, even though the reported accuracy of LUS in this subset of patients was similar to that of the total study population. The low accuracy of CXR might be because a radiogram is usually performed in bed with only an anterior–posterior scan due to the impossibility to move the patients to the radiology ward. The operation of ultrasound examination is strictly dependent on the experience of the operator; however, the image interpretation itself is less dependent on the operator. Based on this assumption in our study, all the images acquisition has been performed by a resident emergency physician, and the interpretation was then reviewed with a senior cardiologist with long experience in LUS. The inclusion of LUS as one of the diagnostic tests to be evaluated in the diagnosis of pneumonia might have led to an incorporation bias which might overestimate the diagnostic accuracy of LUS. However, the accuracy of LUS has been tested also using a CT scan as a reference test and thus avoiding any incorporation bias, confirming substantially the same level of accuracy

## 5. Conclusions

Lung ultrasound is a useful tool for the diagnosis of acute pneumonia in patients admitted in a coronary care unit, performing much better than chest X ray and with slightly lower accuracy than a chest CT scan. This should be considered the method of choice for chest imaging especially when the patients cannot perform a standard chest-X ray with patients standing up in double projection or cannot easily be moved to the radiology ward. Based on our findings, we strongly suggest that positive findings on LUS combined with markers of inflammation should be used for the initial diagnosis and management of acute pneumonia.

## Figures and Tables

**Table 1:** Baseline characteristics of the patients.

	Total Study Population *n* = 110	With Pneumonia *n* = 26	Without Pneumonia *n* = 84	*p* Value
Age (years)	70 ± 11	72 ± 12	69.6 ± 11	0.383
Women (%)	31.8	20	80	0.54
White blood cell count(×10^3^/µL)	10.6 ± 5.4	13 ± 8	9.8 ± 4	0.12
Hemoglobin (g/dL)	12.4 ± 2.2	11.8 ± 2.2	12.6 ± 2.2	0.142
Hs C reactive protein (mg/dL)	44.3 ± 57.9	102 ± 68	24.26 ± 38	0.0001
BNP (pg/mL)	1793 ± 3186	2099 ± 4215	1770 ± 2661	0.799
pH	7.45 ± 0.06	7.46 ± 0.07	7.43 ± 0.05	0.094
PaO_2_/FiO_2_	256 ± 105	220 ± 96	277 ± 106	0.048
Lactate level (mmol/L)	1.4 ± 0.9	1.3 ± 0.6	1.4 ± 1.05	0.575
Pleural effusion (%)	54.8	84.6	39.3	0.0001
Interstitial syndrome (%)	66.7	73.1	65.5	0.470
Concomitant heart failure (%)	49	46.2	50	0.732

**Table 2:** Sensitivity, specificity, positive and negative predictive value, positive and negative likelihood ratio of LUS and CXR (*n* = 110).

	Sensitivity (%) (95% CI)	Specificity (%) (95% CI)	PPV (%) (95% CI)	NPV (%) (95% CI)	Positive Likelihood Ratio	Negative Likelihood Ratio
Lung ultrasound	92.6% (75.7–99.1)	80.95% (70.92–88.70)	61% (48.77–70.27)	97.14% (89.94–99.23)	4.85	0.1
Chest X ray	42.86% (21.82–65.98)	95.12% (87.98–98.66)	69.23% (43.42 86.84)	86.67 (81.73–90.43)	3.95	0.37

**Table 3:** Sensitivity, specificity, and accuracy of LUS and CXR based on presence or absence of pleural effusion.

	Sensitivity (%) (95% CI)	Specificity (%) (95% CI)	PPV (%) (95% CI)	NPV (%) (95% CI)	Accuracy (%)
Panel a No pleural effusion (*n* = 55)					
Lung ultrasound	100% (40–100)	96.1% (86.5–99.5)	67% (34–89)	100% (--)	96.4%
Chest X ray	50% (7–93)	96.1% (86- 99.5)	50% (15.6–84.2)	96.1 (90.1–98.4)	92.7%
Panel b pleural effusion (*n* = 55)					
Lung ultrasound	90.1% (71–98.8)	57.6% (39.2–74.5)	58.5 (48.4–68.5)	98.5 (71–97.3)	72%
Chest X ray	91.8% (14–55)	92.4% (79–98.6)	76% (44.1–92)	66% (58–70%)	64%

**Table 4:** Sensitivity, specificity, positive and negative predictive value, positive and negative likelihood ratio in patients with available all three diagnostic modality (*n* = 34).

	Sensitivity (%) (95% CI)	Specificity (%) (95% CI)	PPV (%) (95% CI)	NPV (%) (95% CI)	Positive Likelihood Ratio	Negative Likelihood Ratio
Lung ultrasound	100% (71.5–100)	73.9% (51.6–90)	64.7% (47.8–78.5)	100% (--)	3.83	-
Chest X ray	18.2% (2.3–51.8)	91.3% (71.2–98.9)	50% (13.9–86.1)	70 (63.2–76.0)	2.09	0.9
Chest CT scan	90.9% (58.72–99.77)	95.6% (78.05–99.89)	90.91 (59.30–98.56)	95.65 (77.21–99.30)	20.9	0.1

**Table 5:** Diagnostic accuracy of LUS to identify lung consolidation compared to chest CT scan (*n* = 34).

	Sensitivity (%) (95% CI)	Specificity (%) (95% CI)	PPV (%) (95% CI)	NPV (%) (95% CI)	Accuracy (%)
Panel a (*n* = 110)					
Lung ultrasound	100% (74–100)	74% (52–89.70)	66.7% (50.1–79.9)	100% (--)	83%

## Data Availability

Not applicable.
